# Adherence to Antiretroviral Therapy and associated factors among HIV infected children in Ethiopia: unannounced home-based pill count versus caregivers’ report

**DOI:** 10.1186/1471-2431-13-132

**Published:** 2013-09-02

**Authors:** Silenat Biressaw, Woldaregay Erku Abegaz, Markos Abebe, Workeabeba Abebe Taye, Mulugeta Belay

**Affiliations:** 1 Tikur Anbessa Hospital, Addis Ababa University, P.O. Box 1176, Addis Ababa, Ethiopia; 2 Aklilu Lemma Institute of Pathobiology, Addis Ababa University, P.O. Box 1176, Addis Ababa, Ethiopia; 3 Armauer Hansen Research Institute, P.O. Box 1005, Addis Ababa, Ethiopia; 4 Department of Pediatrics, School of Medicine, Addis Ababa University, P.O. Box 1176, Addis Ababa, Ethiopia

**Keywords:** Children, HAART, Adherence, Home-based unannounced pill count, Ethiopia

## Abstract

**Background:**

The introduction of Antiretroviral Therapy (ART) has brought a remarkable reduction in HIV-related mortality and morbidity both in adults and children living with HIV/AIDS. Adherence to ART is the key to the successful treatment of patients as well as containment of drug resistance. Studies based on caregivers’ report have shown that adherence to ART among children is generally good. However, subjective methods such as caregivers’ report are known to overestimate the level of adherence. This study determined the rate of adherence and its predictors using unannounced home-based pill count and compared the result with caregivers’ report in a tertiary referral hospital in Ethiopia.

**Methods:**

A cross-sectional study was conducted between December 1, 2011 and January 30, 2012. The study participants were 210 children on ART and their caregivers attending pediatric ART clinic of Tikur Anbessa Hospital (TAH), Addis Ababa University. Caregivers were interviewed at the ART clinic using a structured questionnaire. Then, unannounced home-based pill count was done 7 days after the interview.

**Results:**

Caregiver-reported adherence in the past 7 days prior to interview was 93.3%. Estimated adherence using unannounced home-based pill count was found, however, to be 34.8%. On multivariate logistic regression model, children with married [aOR = 7.85 (95% CI: 2.11,29.13)] and widowed/divorced [aOR = 7.14 (95% CI: 2.00,25.46)] caregivers, those who were not aware of their HIV sero-status [aOR = 2.35 (95% CI:1.09, 5.06)], and those with baseline WHO clinical stage III/IV [OR = 3.18 (95% CI: 1.21, 8.40] were more likely to adhere to their ART treatment. On the other hand, children on d4T/3Tc/EFV combination [OR = 0.10 (95% CI: 0.02, 0.53)] were less likely to adhere to their treatment. Caregivers’ forgetfulness and child refusal to take medication were reported as the major reasons for missing doses.

**Conclusion:**

The level of adherence based on unannounced home-based pill count was unacceptably low. Interventions are urgently needed to improve adherence to ART among children at TAH. Besides, a longitudinal study measuring adherence combined with clinical parameters (viral load and CD4 count) is needed to identify a simple and reliable measure of adherence in the study area.

## Background

HIV/AIDS is one of the most devastating pandemics humanity has ever faced. Globally, about 34 million people were living with the virus in 2010 [1]. In the same year, children contributed to 10% of the total infected. Sub-Saharan Africa remains the most hard-hit region accounting for 68% of the total global burden [1]. Although the prevalence is relatively low, Ethiopia carries one of the largest HIV-infected populations in the world. Out of the 1.1 million people living with HIV, about 72,000 were reported to be children [2].

In an effort to curb the epidemics as well as to improve the quality of life among HIV-infected people, multiple strategies including treatment of patients with ART have been implemented worldwide. The introduction of ART has resulted in a remarkable reduction of HIV-related mortality and morbidity [3-6] as a result of rapid immunological restoration [3,7,8] and viral suppression [3,8].

However, ART provision has major challenges. Despite efforts made over the last decade, universal access to ART especially in low income countries remains low: only half of adults and a quarter of children eligible for ART have started treatment [1]. Among those on ART, retention as well as adherence to therapy remains to be major obstacles for the successful treatment of HIV-infected patients. Adherence to ART is the key to achieving optimal therapeutic effects [9]. Studies indicate that poor adherence is associated with virologic failure [10,11] and increased mortality [12].

Hence, unacceptably high level of virologic failure [10,13,14], limited access to second line drugs in low income countries and the public risk of transmitted resistance as HIV infected children living into adolescence become engaged in sexual relationships make maintaining adherence among children crucial [15]. For optimal therapeutic effect and to minimize HIV drug resistance, patients should have at least ≥ 95% adherence to ART [6,11,16].

Designing strategies for maintaining optimal level of adherence among children is an essential step towards ensuring treatment success. However, this task requires careful assessment of the status of level of adherence and its predictors among the target population [6,17]. In pediatric patients, adherence is more complex as it involves factors related to children, caregivers, family, regimen, society and culture [15,17], and measuring adherence remains a challenge since there is no single method that is reliable and simple [15].

Globally, the level of adherence to ART among HIV infected children varied from 49% to 100% [18] depending on the settings and the methods used. The most frequently used measure of adherence in children is self or caregivers’ report, and the highest level of adherence (79.5-100%) emerged from such measurements [18]. Previous studies among Ethiopian children [19] and adults [20,21] reported a high level of adherence to ART using self /caregivers’ report. However, self or caregivers’ report method alone, although simple, is subjective and subject to social desirability [17] and recall bias. Pill count is a more objective method to assess medication adherence and is recommended as a standard for clinical practice [22]. Therefore, this study looks into the level of adherence and associated factors among pediatric HIV patients taking ART in a pediatric ART clinic using unannounced home-based pill count and compares the result to caregivers’ reported adherence.

## Methods

### Study area

The study was conducted at the pediatric ART clinic of TAH, Addis Ababa University. TAH, located in Addis Ababa, is the single largest referral and tertiary level teaching hospital in Ethiopia. Patients are referred to this hospital from other health institutions across the country. After the government launched free ART in 2005, the hospital started to provide the service to people living with HIV/AIDS including children. The hospital has separate ART clinics for adults and children.

The hospital provides both first line and second line drug regimens. First line regimens that children were taking included 4a = d4T/3Tc/NVP (Stavudine, Lamivudine, Nevirapine), 4b = d4T/3Tc/EFV (Stavudine, Lamivudine, Efavirenz), 4c = AZT/3Tc/NVP (Zidovudine, Lamivudine, Nevirapine), and 4d = AZT/3Tc/EFV (Zidovudine, Lamivudine, Efavirenz). Some children were also on the following second line regimens: ABC/ddi/LPv/r (Abacavir, Didanosine, retonavir enhanced Lopinavir), AZT/3Tc/LPv/r (Zidovudine, Lamivudine, retonavir enhanced Lopinavir) and D4T/3TC/LPv/r (Stavudine, Lamivudine, retonavir enhanced Lopinavir). The only liquid formulation available during the study period was LPv/r and only one child was taking this formulation as a component of D4T/3TC/LPv/r. The routine laboratory tests included CD4 count, haemoglobin and blood chemistry such as liver function tests. Among these, CD4 count was done every 6 months and the rest were done on the basis of clinical conditions of children.

### Study design and study participants

A cross-sectional study was conducted between December 1, 2011 and January 30, 2012. Since the initiation of free ART programme, a total of 1152 HIV positive children were registered at the pediatric ART clinic of the hospital. Of these children, 751 were started on treatment and 554 of them were on active follow-up during data collection. All children (<15 years) on ART for at least three months and their caregivers were consecutively included until sample size was achieved. Children living in an orphanage, those younger than 3 months as well as those living out of Addis Ababa were excluded. Sample size of 210 was calculated using the formula for estimation of single population proportion with 95% CI, 5% margin of error, estimated adherence level of 86.9% [19] and a 20% non-response rate.

### Method of data collection

Data collection was done using a pre-tested, structured questionnaire addressing demographic, socioeconomic and disclosure variables of caregivers and children, as well as health care providers and health care system variables. Nine questions were posed to assess knowledge of caregivers about the ART regimen their children were taking and the median score was taken as a cut-off to classify caregivers as having good or poor knowledge. Available clinical (WHO stage) and immunological (CD4 count) data of children were extracted from records. Caregivers were interviewed during routine clinic appointments in a separate room. Care givers’ home addresses were recorded at the time of interview after getting permission for a home visit; however, caregivers were not informed regarding pill count until home visit was made to avoid bias. Home-based unannounced pill count was conducted 7 days after interview. To make sure caregivers received the prescribed number of pills from the pharmacy, pill count was done immediately after pills were dispensed. Subsequently, caregivers were instructed to use only the currently dispensed pills and not any pills left at home. Pills left at home were counted separately and were not included in calculating adherence rate. Interview and pill count were done by a trained nurse and one of the authors supervised data collection.

### Operational definitions

**Adherence** – Adherence to a medication is generally defined as the extent to which patients take medications as prescribed by their health care providers. In HIV treatment, adherence < 95% is associated with virologic failure [6,11], opportunistic infections and deaths [23], and therefore, in this study a child was said to be adherent if he/she took ≥95% of the prescribed doses for one week prior to the interview or the pill count.

**Caregiver –** Parent/guardian or person in charge of routinely administering antiretroviral drugs to children on ART.

**Good knowledge-** If care givers answer 7–9 questions correctly

**Poor knowledge-** less than 7 questions answered correctly

**Biological parents** - mother or father of the child

**Non- biological parents** - Relatives, or those who adopted the child

**Baseline CD4 count**: CD4 count done when a child started ART

**Current CD count**: CD4 count done within 6 months of data collection

### Data management and analysis

Data were checked manually, computerized using EPiData version 3.0 and exported to SPSS version 20 for analysis. Chi-square test was used to assess association between adherence and independent variables. The percent of adherence was calculated by dividing the number of doses taken in one week by the total number of doses prescribed for that week. Multivariate logistic regression was done to identify factors independently associated with ART adherence. The association of predictor variables with the dependent variable was described by using 95% confidence interval (CI) and adjusted odds ratio (aOR). A p-value of < 0.05 was considered statistically significant.

### Ethics statement

The study was approved by the Institutional Ethical Review Board of Aklilu Lemma Institute of Pathobiology (ALIPB193/2004), Institutional Ethical Review Board of Medical Faculty, Addis Ababa University (085/11/ped) and Armauer Hansen Research Institute and All Africa Leprosy, Tuberculosis and Rehabilitation Training Centre Ethical Review Committee (Po09/12). Written informed consent was obtained from all caregivers and verbal assent was obtained from children who were aware of their sero-status.

## Results

### Socio-demographic characteristics of caregivers and children

A total of 238 primary caregivers were interviewed of whom 28 refused home visit, making the response rate for pill count 88%. Hence, 210 caregivers were included in the final analysis. The median age of caregivers was 38(IQR 30.8-49) years; and the majority (84.8%) of them were females. Of the 210 children, 109 (51.9%) were males and the median age was 11 (IQR 8–13) years, the vast majority (74.8%) being 9 years or older. The majority of the caregivers were literate and with a monthly income less than 30 USD. More than half of the caregivers were biological parents of the children (Table 1). Of the total caregivers, 139(66.2%) have good level of knowledge regarding ART with a median knowledge score of 7(IQR 6–8). All of the caregivers were able to correctly tell the right dose and frequency of pills that their children were taking.

**Table 1:** Socio-demographic characteristics of caregivers and children on ART, TAH, Addis Ababa, 2012

**Characteristics**	**Non-adherent**	**Adherent**	**p-value**
	**number (%)**	**number (%)**	
**Caregivers’ sex**			
Male	20 (62.5)	12 (37.5)	0.77
Female	116 (65.2)	62 (34.8)	
**Caregivers’ age**			
18-40	83 (63.4)	48 (36.6)	
41-65	38 (63.3)	22 (36.7)	
66-90	15 (78.9)	4 (21.1)	0.40
**Caregivers’ marital status**			
Single	24 (82.8)	5 (17.2)	
Married	48 (61.5)	30 (38.5)	0.09
Widowed/divorced	64 ( 62.1)	39 (37.9)	
**Caregivers’ religion**			
Orthodox	97 (66.0)	50 (34.0)	
Muslim	21 (61.8)	13 (38.2)	
Protestant	18 (62.1)	11 (37.9)	0.85
**Caregivers’ education**			
Illiterate	40 (69.0)	18 (31.0)	
Elementary education	53 (65.4)	28 (34.6)	
Secondary and above	43 (60.6)	28 (39.4)	0.60
**Caregivers’ occupation**			
House wife	41 (62.1)	25 (37.9)	
Employed	25 (69.4)	11 (30.6)	0.82
Daily laborer	38 (62.3)	23 (37.7)	
Others**‡**	32 (68.1)	15 (31.9)	
**Monthly income of care givers†**			
<30 USD	100 (64.9)	54 (35.1)	
≥30 USD	36 (64.3)	20 (35.7)	0.93
**Caregivers’ relation to child**			
Biological	76 (62.8)	45 (37.2)	
Non-biological	60 (67.4)	29 (32.6)	0.49
**Age of child**			
1-5	12 (92.3)	1 (7.7)	
6-8	24 (60)	16 (40)	0.09
9-14	100 (63.7)	57 (36.3)	
**Sex of child**			
Male	69 (63.3)	40 (36.7)	
Female	67 (66.3)	34 (33.7)	0.65
**Children knew their HIV status**			
Yes	63 (70.8)	26 (29.2)	
No	73 (57.7)	48 (42.3)	0.11
**Received any support**			
Yes	52 (70.3)	22 (29.7)	
No	84 (61.8)	52 (38.2)	0.218

**†**Exchange rate −1$ = 17.6 Ethiopian birr.

**‡**others: students, daily laborers.

### Social and disclosure variables of children

One hundred and ten (52.4%) caregivers reported that they have disclosed their children’s HIV status to others (71.8% disclosed to the family and 28.2% disclosed to the community) and 89(42.4%) disclosed to the children themselves. However, most (82.9%) of the caregivers were not comfortable to give ART drugs to their children in the presence of others.

Seventy-four (35.2%) of the caregivers had support (mainly as food or cash) for their children from different sources, notably from non-governmental organizations; however, only 6(8.11%) reported that they were satisfied with the support they had received. Regarding the types of regimens children have been taking, 67.6% were either on 4d (AZT/3TC/EFV) or 4c (AZT/3TC/NVP).

### Clinical markers of children on ART

The median length of time on ART was 48 months (IQR: 30–66 months). Based on review of clinical records, 79% of them were either in stage III or Stage IV of WHO classification at baseline. At the time of treatment initiation, median CD4 count was 265 (IQR: 179–461) cells/mm^3^ whereas the current median CD4 count was 617 (IQR: 451–855) cells/mm^3^**.** There was a significant improvement in the current CD4 count compared to the baseline CD4 count (Wilcoxon, p < 001). On stratifying by age, the improvement remained statistically significant [Wilcoxon, p = 0.005 (≤ 5 years) and p < 0.001 (above 5 years)].

### Health care providers and health care system variables

The vast majority (93.3%) of children have been on ART for at least one year. Eighty-one percent of the caregivers reported that they had very good relationship with the health providers at the ART clinic. Furthermore, 206 (98.1%) had open communication and got information needed from health care providers, and 208 (99.1%) had access to the pharmacy at any day of the week. Two hundred and five of them (97.6%) were satisfied in the scheduled appointment and 204 (97.1%) were satisfied with their child’s health improvement.

### Adherence to ART

Based on caregivers’ report, two-third (66.7%) of the study participants had no history of missed doses ever and 90% had no missed doses in the last one month. A total of 196 (93.3%) children were reported to have adherence rate of at least 95% for the past 7 days before interview. However, 96 (47.7%) caregivers reported that medication had been taken several hours late on occasions within the last week.

**Figure 1:**
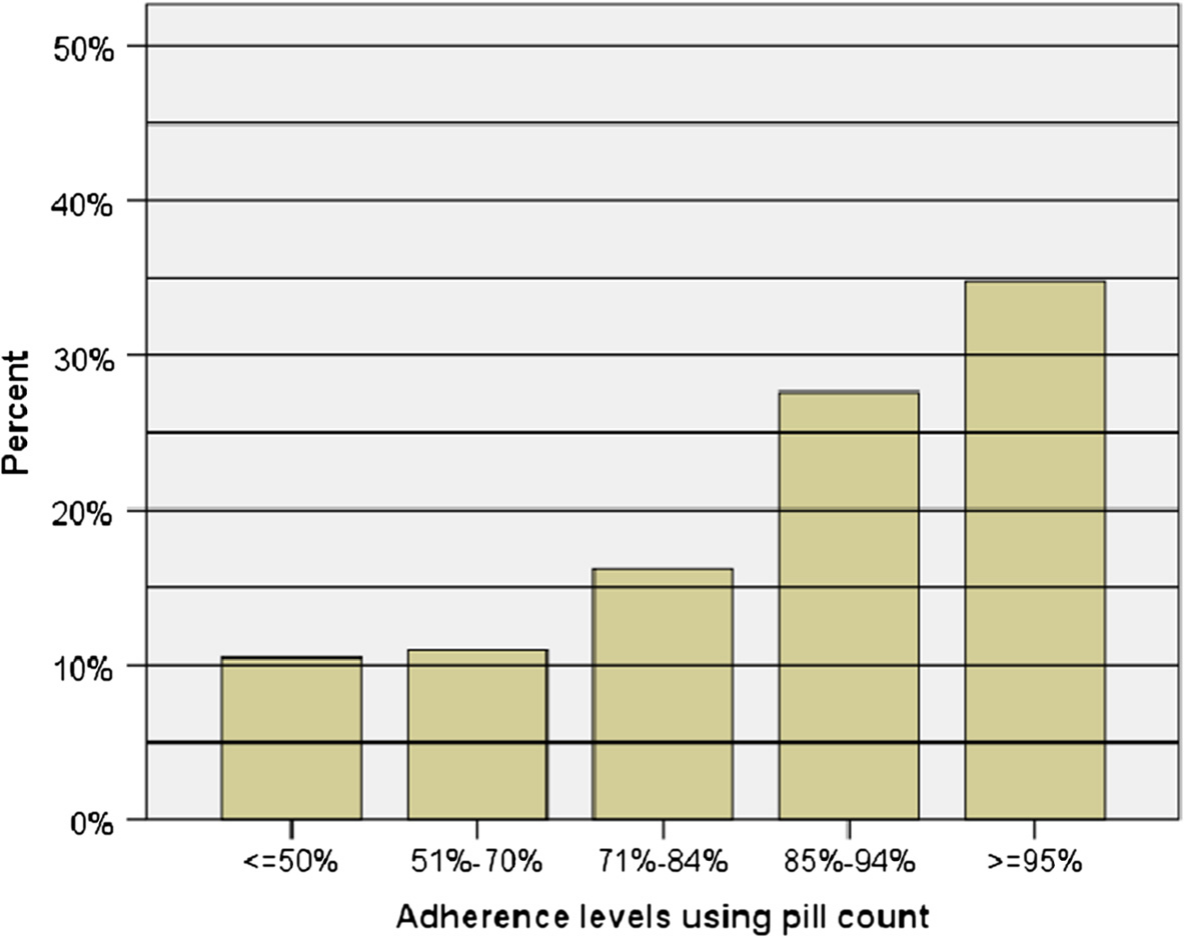
Children’s level of adherence to ART estimated by unannounced home-based pill count, TAH, Addis Ababa, 2012.

On unannounced home-based pill count, only 73 (34.8%) had adherence level of at least 95% which is much less than the reported level of adherence (Figure 1). The level of agreement between caregivers’ report and unannounced pill count was poor (kappa = 0.032).

The median number of doses missed in one week was 1 (IQR: 0–2). Extra pills were counted during home visit and 37.1% of children had no extra pills, whereas 56.3% had 1 to 50 extra doses at home (Table 2). There was no significant difference in the level of adherence between those who had and who did not have extra doses at home (p = 0.46).

**Table 2:** Missed doses, extra doses and adherence on unannounced home-based pill count of children on ART at TAH, Addis Ababa, 2012

**Characteristics**	**Number (%)**
**Doses missed in one week**	
0	73 (34.8)
1–2	90 (42.9)
3–4	30 (14.3)
>4	17 (8.1)
**Extra doses at home**	
0	78 ( 37.1)
1–10	52 (24.8)
11–20	35 (16.7)
21–50	31 (14.8)
>50	14 (6.7)
**Adherence status**	
Non-adherent	137 (65.2)
Adherent	73 (34.8)

**Figure 2:**
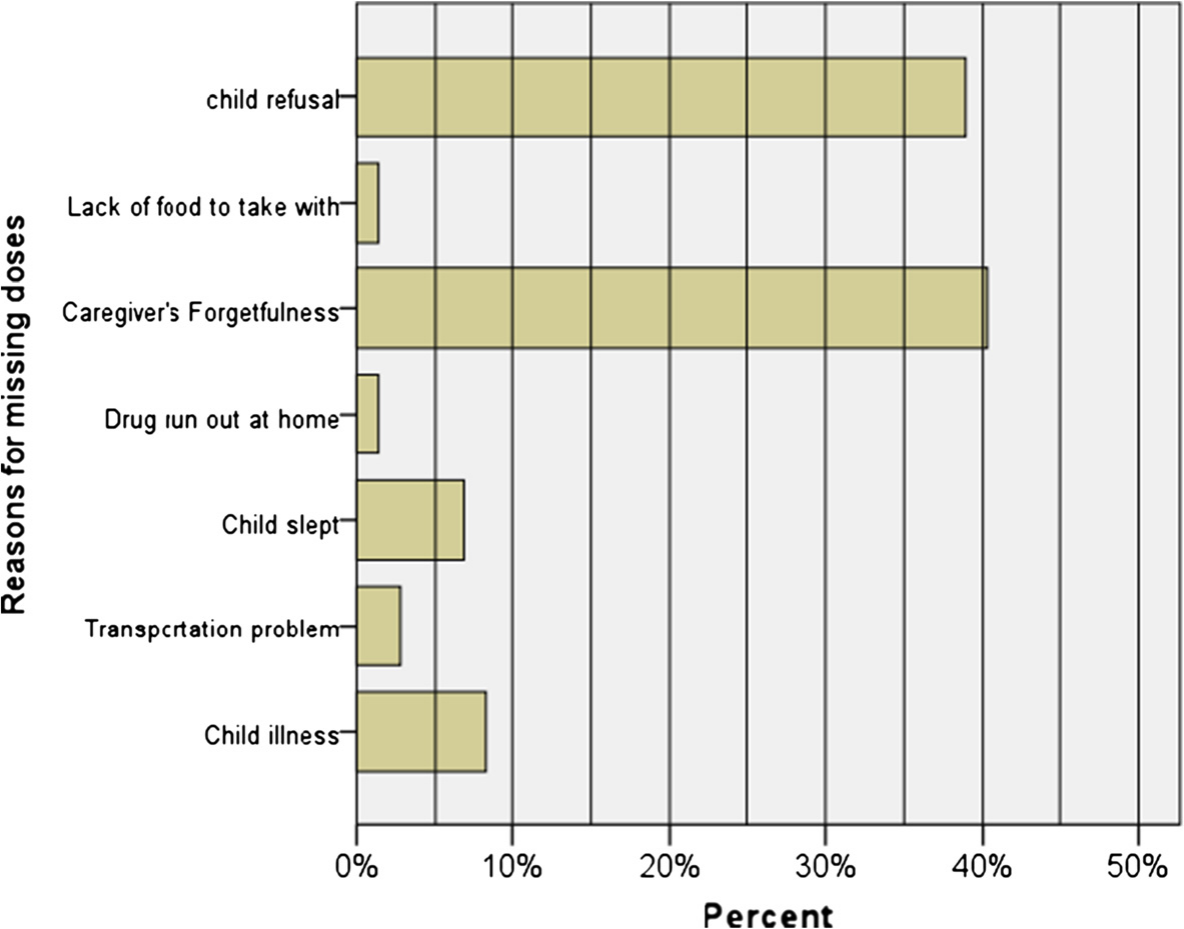
Reasons for missing doses mentioned by care givers of children on ART follow up at Tikur Anbessa Hospital, Addis Ababa, 2012.

When caregivers were asked regarding the type of memory aid they used, the majority (67.1%) reported that they used a watch as memory aid for giving medication. Caregivers’ forgetfulness (36.4%) and child refusal to take medication (27.3%) were reported to be the major reasons for missing doses and delayed ingestion of their pills (Figure 2).

On multivariate logistic regression, children with a married [aOR = 7.85 (95% CI: 2.11, 29.13)] or widowed/divorced caregiver [aOR = 7.14 (95% CI: 2.00, 25.46)] were more likely to adhere to their ART treatment when compared to those with single caregivers. Similarly, children who did not know their HIV sero-status were more likely to adhere to their treatment [aOR = 2.35 (95% CI: 1.09, 5.06)] compared to those who knew their sero-status. Those children with baseline WHO clinical stage III/IV were 2.78 times [OR = 3.18 (95% CI: 1. 21, 8.40)] more adherent to their ART treatment when compared to those with stage I/II. On the other hand, children on d4T/3Tc/EFV combination were less adherent than those who were on d4T/3Tc/NVP combination [OR = 0.10 (95% CI: 0.02, 0.53)] (Table 3).

**Table 3:** Predictors of ART adherence among children at TAH, Addis Ababa, 2012

**Characteristics**	**Non-adherent**	**Adherent**	**Crude OR**	**Adjusted OR**
	**number**	**number**	** (95% CI)**	** (95% CI)**
**Care givers’ marital status**				
Single	24	5	Reference	Reference
Married	48	30	3.00 (1.03, 8.71)	7.85 (2.11, 29.13)*
Widowed/ Divorced	64	39	2.93 (1.03, 8.30)	7.14 (2.00, 25.46)*
**Children knew their HIV status**				
Yes	63	26	Reference	Reference
No	73	48	1.59 (0.89, 2.86)	2.35 (1.09, 5.06)*
**Regimen type**				
4a = d4T/3TC/NVP	26	14	Reference	Reference
4b = d4T/3TC/EFV	17	2	0.22 (0.04, 1.09)	0.10 (0.02, 0.53)*
4c = AZT/3TC/NVP	32	26	1.51 (0.66, 3.46)	1.50 (0.54, 4.13)
4d = AZT/3TC/EFV	53	31	1.09 (0.50, 2.39)	0.63 (0.24, 1.67)
Others$	8	1	0.23 (0.03, 2.05)	0.11 (0.01, 1.23)
**Duration on ART**				
<12 months	13	1	Reference	Reference
≥ 12 months	123	73	7.71 (0.99,60.20)	8.37 (0.91,77.32)
**Baseline WHO stage**				
Stage I and II	34	10	Reference	Reference
Stage III and IV	102	64	2.13 (0.99,4.61)	3.18 (1,21,8.40)*
**Baseline CD4**				
≤200	44	25	Reference	Reference
201–500	60	37	1.09 (0.57,2.06)	0.81 (0.37,1.75)
>500	32	12	0.66 (0.29,1.51)	0.37 (0.13,1.03)

^*^p < 0.05.

*OR*  odds ratio.

*CI*  confidence interval.

Variables included in the logistic regression model were age and sex of child, caregivers’ age, caregivers’ sex, caregivers’ education and income.

$Others: ABC/ddi/LPv/r, AZT/3Tc/LPv/r and D4T/3TC/LPv/r.

## Discussion

Unlike previous studies in Ethiopia, this study determined the level of adherence and its predictors using unannounced home-based pill count and compared the result with caregivers’ report. Based on caregivers’ report, the estimated adherence rate in the last 7 days prior to interview was 93.3%, slightly higher than caregivers’ reported adherence level in a similar setting [19]; however, using unannounced home-based pill count, the adherence rate (34.8%) was unacceptably low. There is an enormous discrepancy between optimal adherence rate (≥95%) estimated by caregivers’ report (93.3%) and unannounced home-based pill count (34.8%). Similar studies elsewhere reported discrepancies between unannounced home-based pill count and caregivers’ report. For example, a study in Uganda among children on ART reported adherence rate of 89% and 94% using self-report and clinic based pill count, respectively; however, on subsequent unannounced pill count, only 72% of children were found to be adherent to their treatment [24]. Similarly, a study in Tanzania [25] among adults on ART revealed that 98% and 93% of patients were adherent based on self-report and hospital pill count, respectively but only 58% were found adherent on unannounced home visit pill count. Another study from South Africa reported that objective methods gave lower levels of adherence [26] implying that subjective methods such as caregivers’ report overestimate levels of adherence.

The level of adherence estimated by unannounced home-based pill count in our study is very low as opposed to many other studies. However, three studies using objective methods like medication event monitoring system (MEMS) reported very low (31-39%) adherence rates [26-28] which are comparable to our findings. The difference in the level of adherence among studies could be explained by differences in the methods used. Almost all studies which used subjective methods reported a high level of adherence compared to those that used objective methods. Studies [26-28] have shown that high levels of adherence as measured by objective methods correlate well with virologic suppression showing that objective methods might reflect the actual level of adherence.

Children skip doses for a number of reasons. In this study, caregivers’ forgetfulness was reported as a major reason (36.4%) for skipping doses which is in agreement with other similar studies [19,29]. The vast majority of the caregivers in our study were poor and amidst multiple responsibilities in meeting daily life demands, they could easily forget to make sure their children have taken their pills as scheduled. Moreover, child refusal to take medication (27.3%) and child illness (12.1%) were reported to be the other major reasons for skipping doses. These same reasons were mentioned in a review of studies among adults and children [6].

A number of factors have been reported as predictors of non-adherence among children on ART. In this study, children with married and widowed/divorced caregivers were significantly more adherent when compared to children with single caregivers, which is in agreement to a report from Nigeria [30]. Married caregivers might get support from their spouse in providing care to their children. Besides, widowed and divorced caregivers might be more experienced in handling children as compared to single caregivers.

The association between awareness of sero-status and adherence remains controversial. In this study like many other studies [19,31,32], children who were aware of their HIV status were less adherent than those who were not. However, other studies [15] reported that lack of disclosure of their HIV sero-status among children predicted non- adherence. Children who are aware of their sero-status might deny, feel hopelessness and skip treatment doses until they accept their HIV sero-status; and the observed difference across studies might be related to the extent and duration of counseling offered to children. Those children who have been getting regular counseling and support might accept their sero-status and be more adherent to their treatment than those who do not get proper counseling. Nevertheless, investigation of the importance of HIV sero-status disclosure to children is essential to settle these contradictory results.

The association of clinical condition of children at initiation of ART and treatment adherence is not well established. Some studies found an association between worse adherence and advanced disease [15] which is contrary to our finding. In this study, children who were in WHO stage III or IV at baseline were more adherent to their medication compared to those in stage I or II. A study from Uganda reported that those children who were hospitalized twice or more prior to starting ART had better treatment adherence levels [24]. Those children with advanced disease might be more eager to get well and this might be a motive for better adherence to their treatment.

Children taking d4T/3Tc/EFV combination were less likely to adhere than those children on d4T/3Tc/NVP combination. Although every drug has its own adverse effect, lower adherence on children on Efavirenz combination in this study is probably due to its serious side effects including neuropsychological symptoms, such as bad dreams, dizziness, depression, anxiety, hallucinations, aggressive behavior, and suicidal ideation [33].

A cross-sectional study design was employed in this study and its limitations especially recall bias should be appreciated. Besides, participants were included consecutively when they came for their regular clinic appointment and this might have introduced selection bias. This study was done in a season of holidays that brings families together. Caregivers might be reluctant to give medications to their children in front of others to avoid stigma and this might have underestimated the level of adherence. In this study, it was not possible to assess the clinical relevance of adherence among children. Besides, a point adherence level may not reflect the actual long term adherence, implying the need for longitudinal studies.

## Conclusion

In this study, there was a major difference in the level of adherence estimated by caregivers’ report and unannounced home-based pill count. The level of adherence estimated by unannounced home-based pill count was found to be unacceptably low. Interventions towards improving adherence to ART among children at TAH are urgently needed. Moreover, children with single caregivers, those who were aware of their sero-status, those with baseline WHO clinical stage I/II and those on d4t/3TC/EFV combination need special attention to improve their level of adherence to their medication.

A longitudinal study measuring adherence combined with clinical parameters (viral load and CD4 count) is needed to identify a simple and reliable measure of adherence in the study area.

## Competing interests

The authors declare that they have no competing interests.

## Authors’ contributions

SB was involved in the design, data collection and analysis. MB conceived the study, participated in its design & analysis and drafted the manuscript. WEA participated in the design of the study and data analysis. MA and WAT were involved in the acquisition of data. All authors have critically reviewed and approved the final manuscript.

## Pre-publication history

The pre-publication history for this paper can be accessed here:

http://www.biomedcentral.com/1471-2431/13/132/prepub
